# Gut microbiome profiles associated with steatosis severity in metabolic associated fatty liver disease

**DOI:** 10.20517/2394-5079.2021.55

**Published:** 2021-05-08

**Authors:** Tien S. Dong, Kayti Luu, Venu Lagishetty, Farzaneh Sedighian, Shih-Lung Woo, Benjamin W. Dreskin, William Katzka, Candace Chang, Yi Zhou, Nerea Arias-Jayo, Julianne Yang, Aaron I. Ahdoot, Jason Ye, Zhaoping Li, Joseph R. Pisegna, Jonathan P. Jacobs

**Affiliations:** 1 The Vatche and Tamar Manoukian Division of Digestive Diseases, Department of Medicine, David Geffen School of Medicine at UCLA, Los Angeles, CA 90095, USA.; 2 UCLA Microbiome Center, David Geffen School of Medicine at UCLA, Los Angeles, CA 90095, USA.; 3 Division of Gastroenterology, Hepatology and Parenteral Nutrition, Veterans Administration Greater Los Angeles Healthcare System, Los Angeles, CA 90073, USA.; 4 Department of Medicine, Veterans Administration Greater Los Angeles Healthcare System, Los Angeles, CA 90073, USA.; 5 Center for Human Nutrition, David Geffen School of Medicine at UCLA, Los Angeles, CA 90095, USA.

**Keywords:** Metabolic syndrome, nonalcoholic fatty liver disease, microbiome, obesity, ultrasound elastography, advanced steatosis, diabetes

## Abstract

**Aim:**

The microbiome has been shown to be pivotal in the development of metabolic associated fatty liver disease (MAFLD). Few have examined the relationship of the microbiome specifically with steatosis grade. Therefore, our aim was to characterize the association of the microbiome with MAFLD steatosis severity while adjusting for metabolic comorbidities including diabetes.

**Methods:**

We enrolled patients with MAFLD at the West Los Angeles Veterans Affair Hospital. All patients underwent ultrasound elastography, fasting serum collection, and fecal sampling for 16S sequencing. We examined the associations of microbial diversity and composition with advanced steatosis, defined as a CAP score of ≥ 300 dB/m, with or without the presence of metabolic comorbidities.

**Results:**

Seventy-five patients were enrolled. African American were less likely to have advanced steatosis than either Hispanics or Whites (*P* = 0.001). Patients with more advanced steatosis had higher fasting serum triglyceride (192.6 ± 157.1 mg/dL *vs*. 122.5 ± 57.4 mg/dL), HbA1c (6.7% ± 1.4% *vs*. 6.1% ± 0.8%), transaminases, and were more likely to have metabolic syndrome (52.4% *vs*. 24.2%, *P* = 0.02). Advanced steatosis and diabetes were associated with altered microbial composition. *Bacteroides* was negatively associated with advanced steatosis while *Megasphaera* was positively associated with steatosis. *Akkermansia* was negatively associated with diabetes, while *Anaerostipes* and *Parabacteroides* were positively associated with diabetes.

**Conclusion:**

Diabetes and metabolic syndrome are associated with hepatic steatosis severity in MAFLD patients and both advanced steatosis and comorbid diabetes are independently associated with microbiome changes. These results provide insight into the role of the gut microbiome in MAFLD associated with metabolic syndrome.

## INTRODUCTION

Obesity is a rapidly growing epidemic in the United States. It is now estimated that 1 out of every 4 Americans is obese^[1]^. With the rise of obesity, there has also been a rise in obesity-related diseases such as cardiovascular disease, hyperlipidemia, diabetes, and metabolic associated fatty liver disease (MAFLD)^[2,3]^. MAFLD is estimated to affect up to 30%−50% of all obese patients^[4]^. It is projected to be the leading cause of cirrhosis and liver transplant in the next 10 years^[4]^. Despite the rising tide of MAFLD incidence, there are currently no approved medications for the treatment or prevention of MAFLD. While diet and exercise remain pivotal to the treatment of MAFLD, only 10%−15% of patients are able to reach and sustain significant weight loss to affect MAFLD progression^[5]^. Therefore, continued research in this field is paramount to the development of novel therapeutics.

One area that has shown promise in the field of MAFLD and obesity is the gut microbiome. The intestinal microbiome is a complex community of trillions of bacteria with over 2 million genes that acts as a bridge between the environment and the host^[6]^. Over the last decade, the gut microbiome has been shown to play a pivotal role in the development of obesity, insulin resistance, MAFLD, and liver fibrosis^[7–11]^. Germ-free mice have lower body fat and are more resistant to obesity than conventionally housed mice^[12]^. By using fecal microbial transplant, the obesity phenotype has been shown to be transferrable via the gut microbiome^[13]^. Yet, the exact mechanism by which the gut microbiome modulates obesity and MAFLD is still unclear. Several studies have shown associations of bacterial taxa with MAFLD development and progression to fibrosis as compared to healthy controls^[14]^. However, MAFLD is not a singular disease. Instead, it is a disease that is often associated with several other metabolic related diseases such as hypertension, diabetes, high cholesterol, and metabolic syndrome. Very few studies have examined the association of the gut microbiome in patients with MAFLD while accounting for other metabolic co-morbidities. Therefore, the aim of our study was to examine the relationship of the gut microbiome to MAFLD severity while adjusting for other metabolic-related comorbidities.

## METHODS

### Patient recruitment

Patients were recruited at the West Los Angeles Veterans Affair Medical Center. Inclusion criteria included patients between 20–75 years of age and a diagnosis of MAFLD either by imaging or physician notes. Exclusion criteria included having active viral hepatitis, autoimmune hepatitis, or other forms of chronic liver disease, recent weight loss of > 10 kg or were on a calorie-restricted diet 6 months prior to enrollment, abnormal baseline labs (serum creatinine > 1.6 mg/dL; triglycerides > 500; thyroid stimulating hormone outside of normal range), consumption of more than 1 alcoholic beverage per day, or pregnant. Patients were also excluded if they were on antibiotics or probiotics within 1 month of enrollment. At the time of recruitment, clinical data, demographic data, current medications, fecal sampling, fasting serum, and ultrasound elastography were obtained. Ultrasound elastography was done using the FibroScan platform (Echosens, Waltham, MA, USA) with the XL probe by trained technicians with at least 100 prior procedures. Advanced fibrosis was defined as having at least F3 fibrosis on ultrasound elastography and advanced steatosis was defined as having a CAP score of at least 300 dB/m^[15]^. Metabolic syndrome was defined using the National Cholesterol Education Program (NCEP) Adult Treatment Panel III (ATP III) definition^[16]^. Metabolic syndrome was defined as having any three of the following five criteria: waist circumference > 40 inches in males or 35 inches in females, fasting glucose ≥ 100 mg/dL or history of diabetes mellitus on therapy, triglycerides ≥ 150 mg/dL or on therapy for hypertriglyceridemia, high density lipoprotein (HDL) < 40 mg/mL in males or < 50 mg/dL in females or was on therapy to raise their HDL, or blood pressure > 130 mmHg systolic or > 85 mmHg diastolic or was on therapy for hypertension^[16]^. All research was performed in accordance with the Declaration of Helsinki and approved VA IRB. Project identification code: 2017–121121. Written and verbal informed consent was obtained from each participant.

### Fecal sampling and processing

Participants were given a home stool kit and were asked to return the stool sample within 1 week of the patient recruitment. Stool samples were collected in Para-Pak collection vials prefilled with 95% ethanol. Samples were then stored at −80 °C until they were all processed together as a single batch. DNA was extracted using the ZymoBIOMICS DNA Microprep Kit (Zymo Research, Irvine, CA, USA) per the manufacturer’s protocol. Sequencing of the 253 base pair V4 region of 16S ribosomal DNA was performed as previously described using the Illumina NovaSeq 6000 to a depth of 250,000 reads per sample (primer set: 515f/806r)^[17]^. The sequences were processed with the DADA2 pipeline in R which assigns taxonomy using the SILVA 132 database. After pre-processing in R, the data were imported into QIIME 2 version 2019.10 for further analysis. Amplicon sequence variants were filtered in not present in at least 25% of all samples. Sequence depth ranged from 106,156–511,477 with a median value of 224,733.

### Statistical analysis

Baseline clinical and demographic characteristics were compared between patients with severe steatosis (CAP score ≥ 300 dB/m) *vs*. those with lower level of steatosis using analysis of variance for continuous variables and Fisher exact test for categorical variables. For microbiome analysis, continuous laboratory values were dichotomized into categories. The following standard clinical cutoffs were used: total cholesterol: 200 mg/dL, triglyceride: 150 mg/dL, HDL: 40 mg/mL in males or 50 mg/dL in females, LDL: 130 mg/dL, HbA1c: 6.5%, aspartate aminotransferase: 35 IU/L, and alanine aminotransferase: 40 IU/L. Similarly, age and BMI were dichotomized into a binary categorical variable based on the median value of the cohort, 58 for age and 34.1 for BMI. All means were presented with their standard deviations. A composite category of race and ethnicity was used where we identified Hispanics as a separate category (i.e., White, Asian, African American, Hispanic, and others).

Microbiome data were analyzed similar to our previous published works^[11,17]^. Alpha diversity was calculated using the Shannon index, which measures species richness and evenness, with data rarefied to 106,155 sequences. The difference in alpha diversity was calculated using analysis of variance. Beta diversity was determined by using the DEICODE plugin in QIIME2 which uses a robust Aitchison distance metric. This newer form of distance metric has been shown to yield higher discrimination for human studies when compared to UniFrac or Bray-Curtis^[18]^. Statistical significance of beta diversity was determined using the “adonis” package in R (version 4.0.3, Vienna, Austria) which employs a permutational multivariate analysis of variance. Differential abundance testing between groups was assessed using DESeq2 in R which utilizes empirical Bayesian approach to shrink dispersion and fit non-rarified count data to a negative binomial model^[19]^. *P*-values were converted to Q-values to correct for multiple hypothesis testing using a threshold of Q < 0.05 for significance.

## RESULTS

Seventy-five MAFLD patients were enrolled into the study. The average age was 56.0 ± 1.2 years old with a majority of the patients being male patients [Table 1]. There was no statistical difference by age, BMI, or gender between patients with high level of steatosis *vs*. those with lower level of steatosis. Patients with more advanced steatosis were more likely to be White or Hispanic and less likely to be African American (81.0% *vs*. 16.7%, *P* = 0.001). Not surprisingly, patients with more advanced steatosis had higher fasting serum triglyceride (192.6 ± 157.1 mg/dL *vs*. 122.5 ± 57.4 mg/dL), HbA1c (6.7% ± 1.4% *vs*. 6.1% ± 0.8%), aspartate aminotransferase (28.4 ± 13.2 IU/L *vs*. 21.1 ± 6.5 IU/L), and alanine aminotransferase (43.0 ± 23.1 IU/L *vs*. 28.0 ± 15.8 IU/L) as compared to those with lower levels of steatosis [Table 1]. Consistent with the laboratory data, patients with advanced steatosis were more likely to have metabolic syndrome than those without advanced steatosis (52.4% *vs*. 24.2%, *P* = 0.02). Only 11 patients (14.7%) had advanced fibrosis and there was no significant association between the level of steatosis and the presence of advanced fibrosis.

**Figure 1.**
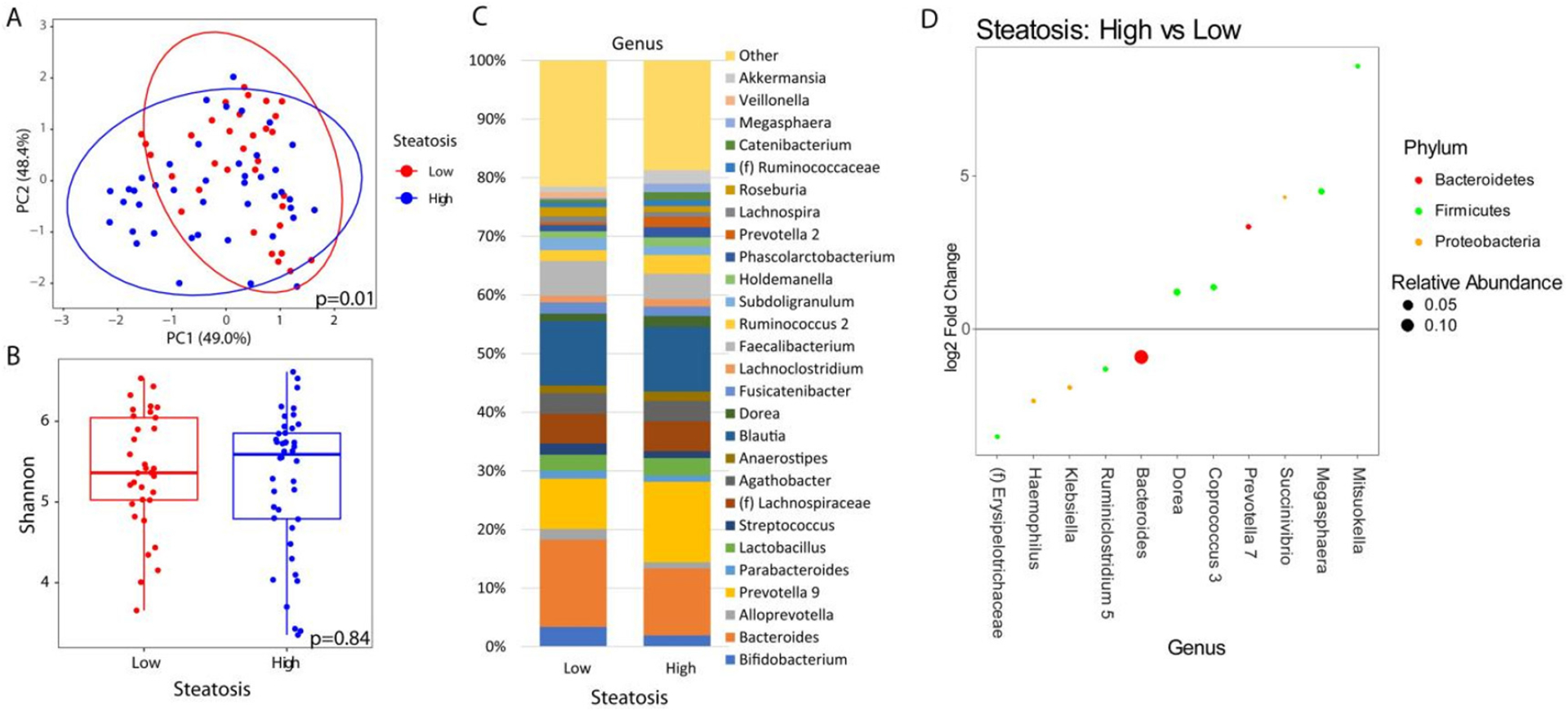
Microbial changes by advanced steatosis. Principal coordinate plot of microbiome data grouped by the presence of high (CAP ≥ 300 dB/m) steatosis or low steatosis (CAP < 300 dB/m). Ellipsis represent 95% confidence interval (A). Alpha diversity as measured by Shannon index between patients with high or low steatosis (B). Taxonomic plot of genera present in patients with high or low steatosis. Only genera with a relative abundance ≥ 1% are shown (C). Differential abundance testing by DESeq2 showing genera that are significantly different between patients with high steatosis as compared to those with low steatosis (D).

**Figure 2.**
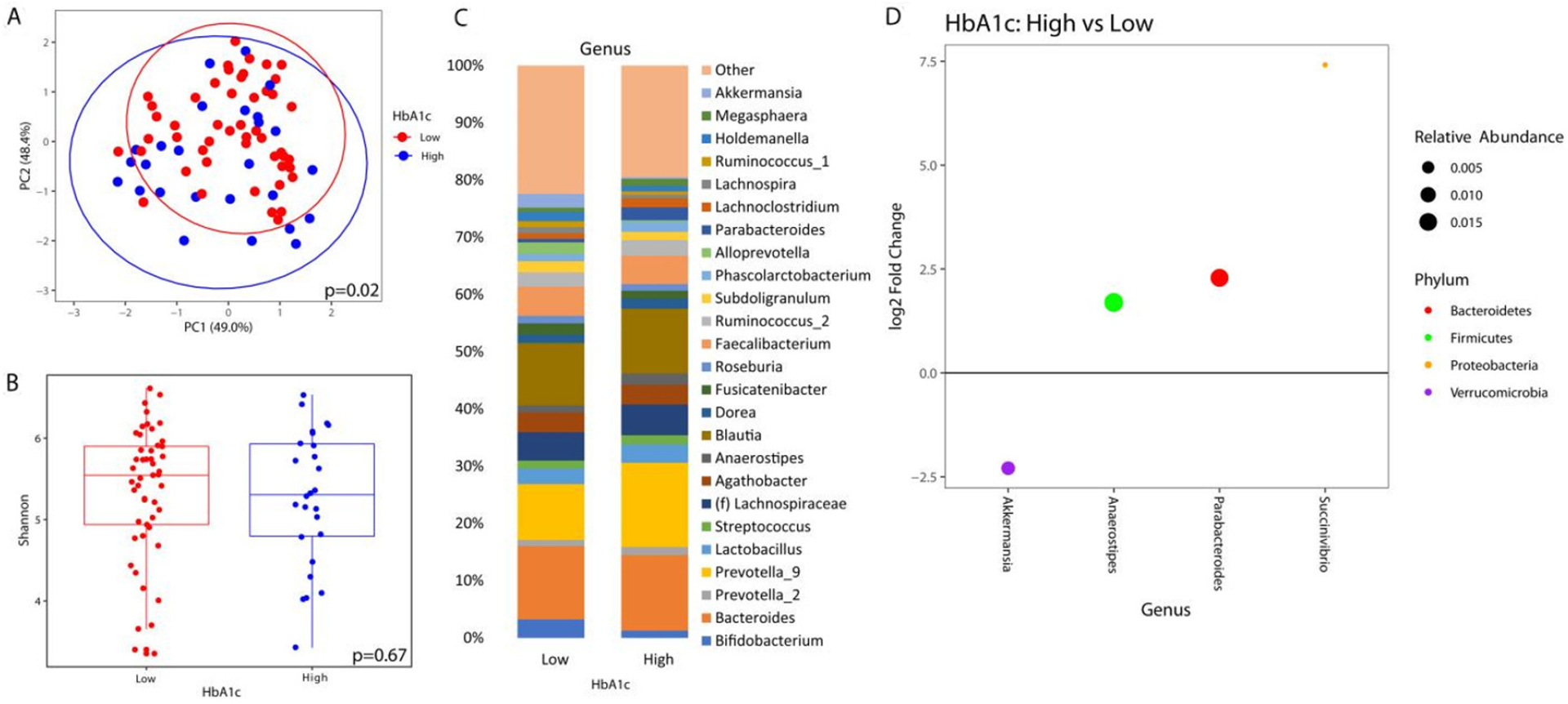
Microbial changes by elevated HbA1c. Principal coordinate plot of microbiome data grouped by the presence of elevated (≥ 6.5%) HbA1c or low HbA1c (< 6.5%). Ellipsis represent 95% confidence interval (A). Alpha diversity as measured by Shannon index between patients with high or low HbA1c (B). Taxonomic plot of genera present in patients with high or low HbA1c. Only genera with a relative abundance ≥ 1% are shown (C). Differential abundance testing by DESeq2 showing genera that are different between patients with elevated HbA1c as compared to those with low HbA1c (D).

When analyzing the fecal microbiome, there were no significant differences in beta diversity by any factor except for the level of steatosis (*P* = 0.01) [Figure 1A] and HbA1c elevation (≥ 6.5%) (*P* = 0.02) [Figure 2A]. A complete list of variables tested by beta diversity and their respective *P*-value is provided in the supplemental tables supplemental tables. There was no difference in alpha diversity by steatosis [Figure 1B]. The genus level composition of subjects by steatosis severity is summarized in Figure 1C. Differential abundance testing showed 11 genera that were different between patients with advanced steatosis and those with less advanced steatosis [Figure 1D]. Five genera were underrepresented while 6 genera were overrepresented in patients with advanced steatosis. Of the 5 underrepresented genera, the genera with the largest relative abundances were *Bacteriodes*, *Ruminoclostridium*, and *Klebsiella*. Of the 6 overrepresented genera, the genera with the largest relative abundances were *Dorea*, *Megashaera*, and *Coprococcus*.

Similarly, there was no significant difference in alpha diversity by HbA1c [Figure 2B]. The taxonomic summary by HbA1c level is shown in Figure 2C. Differential abundance testing showed 4 genera that were different between patients with a HbA1c ≥ 6.5% *vs*. those below 6.5% [Figure 2D]. *Akkermansia* was the only genus that was underrepresented in patients with elevated HbA1c, while *Anaerostipes*, *Parabacteroides*, and *Succinivibrio* were overrepresented.

**Figure 3.**
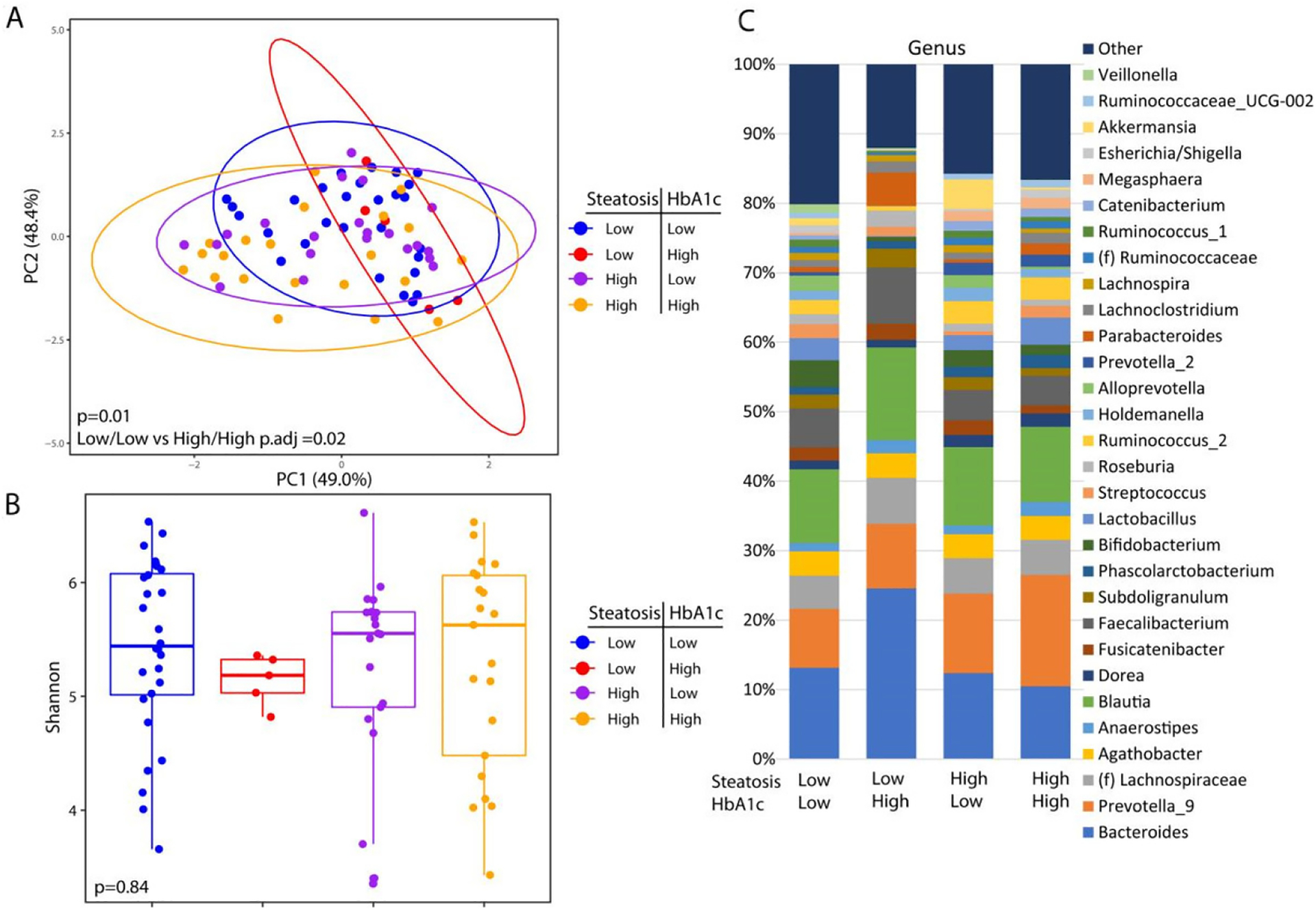
Microbial changes by advanced steatosis and diabetes status. Principal coordinate plot of microbiome data grouped by the presence advance steatosis and/or elevated HbA1c. Ellipsis represent 95% confidence interval (A). Alpha diversity as measured by Shannon index between patients with advance steatosis and/or elevated HbA1c (B). Taxonomic plot of genera present in patients with advance steatosis and/or elevated HbA1c. Only genera with a relative abundance ≥ 1% are shown (C).

**Figure 4.**
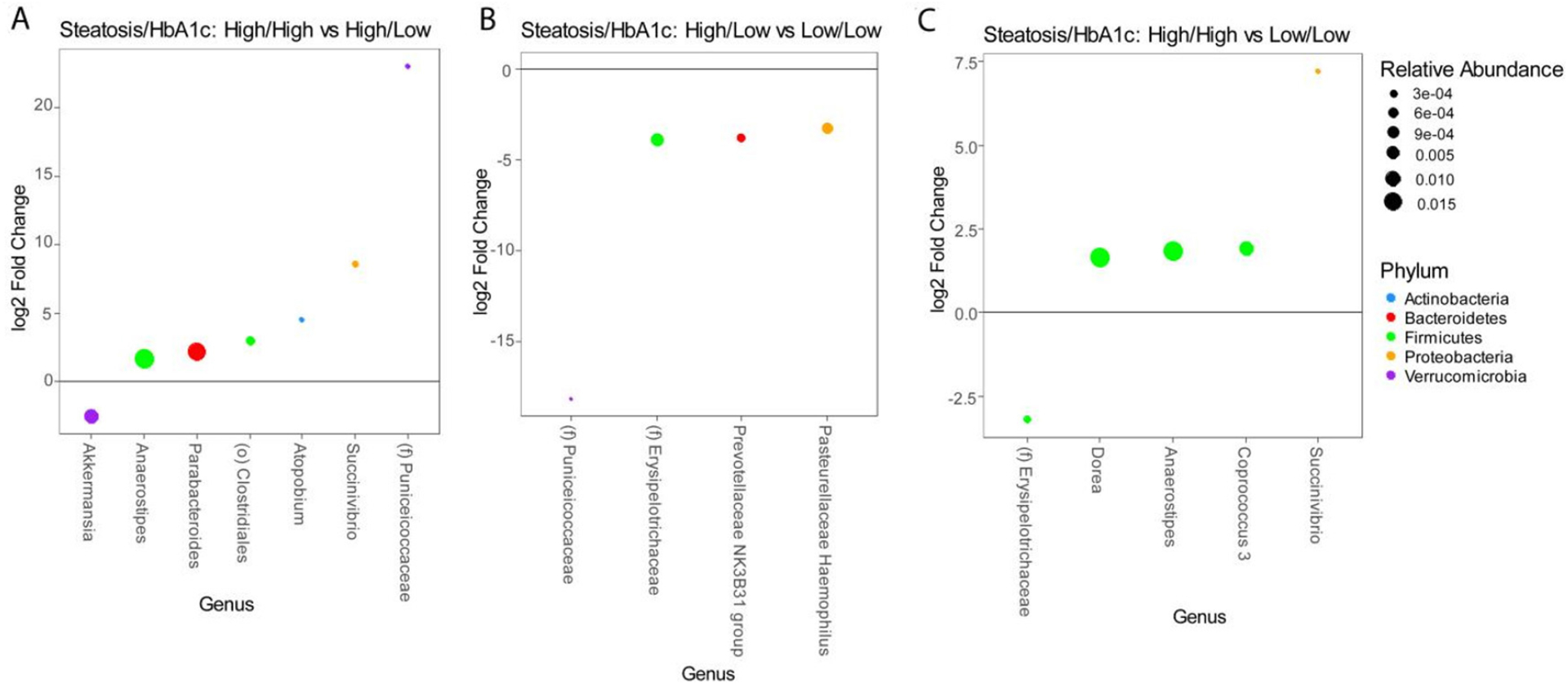
Differential abundance testing by DESeq2 showing genera that are different between (A) patients with advanced steatosis with or without elevated HbA1c, (B) patients without elevated HbA1c with or without advanced steatosis, and (C) patients with both advanced steatosis and elevated HbA1c as compared to those with neither.

These analyses are confounded by the association of diabetes with advanced steatosis. In order to characterize the independent associations of steatosis and diabetes with the gut microbiome, we stratified patients into four groups based on the presence or absence of advanced steatosis and elevated HbA1c. There were 28 patients without advanced steatosis and without an elevated HbA1c, 21 patients with advanced steatosis with elevated HbA1c, 21 patients with advanced steatosis without an elevated HbA1c, and only 5 patients who had an elevated HbA1c without evidence of advanced steatosis. The microbiome differed significantly among these 4 groups [Figure 3A] with the greatest difference between patients with both advanced steatosis and an elevated HbA1c as compared to those with neither. There was no difference in alpha diversity across these four groups [Figure 3B] and the taxonomic summary of these groups is represented in Figure 3C. Due to the low number of patients with an elevated HbA1c without advanced steatosis, we excluded this group from differential abundance testing. Comparing patients with advanced steatosis with or without an elevated HbA1c, 7 genera were differentially abundant [Figure 4A]. Only *Akkermansia* was underrepresented in patients with advanced steatosis and an elevated HbA1c as compared to those with advanced steatosis and normal HbA1c. Of the 6 genera that were overrepresented, *Anaerostipes*, *Parabacterioides*, and an unidentified genus belonging to the order *Clostridiales* had the highest relative abundances. Comparing patients with normal HbA1c, patients with advanced steatosis had 4 genera that were underrepresented as compared to those with less advanced steatosis [Figure 4B]. These 3 most abundant genera were *Prevotellaceae NK3B31* group, *Pasteurellaceae Haemophilus*, and a genus belonging to the family *Erysipelotrichaceae*. Comparing patients who had both elevated HbA1c and advanced steatosis to those that had neither, there were 5 genera with differential abundance [Figure 4C]. The only genus that was underrepresented in patients that had both conditions was a genus belonging to the family *Erysipelotrichaceae*. Of the other 4 genera that were overrepresented, the genera with highest relative abundances were *Dorea*, *Anaerostipes*, and *Coprococcus*.

While there was no difference is alpha diversity by either HbA1c or steatosis, we did find alpha diversity difference by age and race. Patients who were older had higher diversity than those who were younger, and Hispanics had the highest diversity. This data are represented in Supplementary Figure 1.

## DISCUSSION

This is one of the few studies to examine the relationship of the gut microbiome with steatosis severity while accounting for other factors associated with metabolic syndrome. Our data suggest that the gut microbiome is altered by both the presence of diabetes and advanced steatosis, and that bacterial shifts are most significant for those that have both as compared to those that have neither.

It is not surprising to see advanced steatosis by ultrasound elastography associated with worsening metabolic syndrome and transaminase elevation. Insulin resistance is often a major driver of MAFLD, and MAFLD is a major driver of insulin resistance^[20]^. The pathophysiology of these two conditions is intertwined and can be difficult to separate. Studies have shown that the presence of metabolic syndrome in patients with MAFLD places them at an increased risk of developing liver fibrosis, cirrhosis, and hepatocellular carcinoma^[21]^. In addition to metabolic syndrome, we also found associations of race and ethnicity with advanced steatosis. In particular, African Americans had a lower risk of having advanced steatosis than Hispanic or non-Hispanic Whites. This is consistent with other studies that have shown a lower risk for MAFLD in African American patients^[22]^. Population-based studies suggest that the prevalence of MAFLD in Hispanics is as high as 1 in 4 while in African Americans it is 1 in 10^[22]^. The reason for this disparity is still unclear, which has been attributed to such factors as genetics, cultural differences, and eating behaviors.

From a microbiome standpoint, we identified several bacterial taxa that were associated with advanced steatosis and diabetes in MAFLD patients. In our study, *Bacteroides* was negatively associated with advanced steatosis and *Megasphaera* was positively associated with advanced steatosis. *Dorea* and *Succinivibrio* were positively associated with both advanced steatosis and diabetes. The role of *Bacteroides* in MAFLD is complex. Some species of *Bacteroides* have been associated with progression to advanced liver fibrosis^[23]^ while other *Bacteroides* species have been reported to be protective against MAFLD^[24]^. *Megasphaera* and *Dorea*, on the other hand, have been consistently associated with advanced MAFLD and steatohepatitis^[25,26]^. Potential mechanisms by which changes in the gut microbiome may affect steatosis include alterations in bile acid signaling, short chain fatty acid signaling, and endotoxemia^[25,27–30]^.

Of the genera that were associated with elevated HbA1c, *Akkermansia*, *Anaerostipes*, and *Parabacteroides* were associated with elevated HbA1c independent of MAFLD. *Akkermansia* is one of the most well-studied bacterial genera in relation to obesity. It is a mucin-degrading bacteria that has been inversely associated with diabetes, obesity, and MAFLD^[31–33]^. In a mouse model, oral administration of *Akkermansia* activated toll-like receptor 2, increased the expression of epithelial tight-junction proteins, and reversed high-fat diet-induced insulin resistance^[34]^. The beneficial role of *Akkermansia* has led several researchers to investigate *Akkermansia* supplementation as a means to treat obesity^[32]^. Similarly, we show that *Akkermansia* was negatively associated with elevated HbA1c. Unlike *Akkermansia*, less is known about the relationship of *Anaerostipes* and *Parabacteroides* with diabetes. In a genome wide association study of human stool sample, researchers found *Anaerostipes* to be positively associated with type 2 diabetes. Similarly, in studies of type 1 diabetes and gestational diabetes, *Parabacteroides* was elevated compared to healthy controls^[35,36]^. Whether these associations are causal is still an area of active research.

Despite the nuance of evaluating the association between the gut microbiome and steatosis while accounting for other metabolic diseases, this study has several limitations. The first is that the study was performed at a Veterans Affair (VA) hospital. While the VA population has a higher prevalence of metabolic diseases and obesity than the general population^[37]^, it is predominantly male. Therefore, the results may be difficult to generalize to MAFLD in other populations. Secondly, while we did see differences in the gut microbiome in patients with diabetes as compared to those without diabetes, we could not account for the effect of metformin use on the microbiome. Metformin has been shown to affect the microbiome independent of diabetes^[38,39]^. However, because nearly all of our patients who had diabetes were on metformin, we were unable to parse out the effect of metformin use from diabetes in our analysis. Lastly, while this study offers new insight into the role of the microbiome in hepatic steatosis and diabetes, due to its cross-sectional design it can only show association rather than causation. Further longitudinal human studies and animal model experiments will be required to further validate these findings and determine potential mechanisms.

In conclusion, in this study, it’s shown that diabetes and metabolic syndrome are associated with hepatic steatosis severity in patients with MAFLD and that these differences are reflected in alterations of the gut microbiome. These findings support further investigation into mechanisms by which the microbiome promotes both hepatitis steatosis and insulin resistance, which may elucidate novel therapeutic targets for diabetes, obesity, and MAFLD.

## Supplementary Material

Supplementary Figure 1

Supplementary Tables

## Figures and Tables

**Table 1.** Patient characteristics

	Low steatosis (*n* = 33)	High steatosis (*n* = 42)	*P*-value
Age	55.3 (10.3)	56.6 (11.2)	0.59
BMI	33.7 (3.7)	35.5 (5.0)	0.08
**Gender (*n*, %)**			
Male (*n* = 61)	24 (39.3%)	37 (60.7%)	0.14
Female (*n* = 14)	9 (64.3%)	5 (35.7%)	
**Race/Ethnicity (*n*, %)**			
African American (*n* = 25)	18 (72.0%)	7 (28.0%)	**0.001**
Asian (*n* = 2)	1 (50%)	1 (50%)	
Hispanic (*n* = 16)	6 (37.5%)	10 (62.5%)	
Other (*n* = 1)	1 (100.0%)	0 (0.0%)	
White (*n* = 31)	7 (22.6%)	24 (77.4%)	
**Laboratory values (mean, standard deviation)**			
Cholesterol (mg/dL)	162.8 (34.4)	178.4 (49.1)	0.14
Triglyceride (mg/dL)	122.5 (57.4)	192.6 (157.1)	**0.02**
HDL (mg/dL)	43.1 (9.2)	41.9 (12.7)	0.65
LDL (mg/dL)	95.6 (33.6)	101.0 (37.0)	0.52
HbA1c	6.1 (0.8)	6.7 (1.4)	**0.03**
AST (IU/L)	21.1 (6.5)	28.4 (13.2)	**0.01**
ALT (IU/L)	28.0 (15.8)	43.0 (23.1)	**0.003**
**Advanced fibrosis (*n*, %)**			
No (*n* = 64)	31 (48.4%)	33 (51.6%)	0.1
Yes (*n* = 11)	2 (18.2%)	9 (81.8 %)	
**Metabolic syndrome (*n*, %)**			
No (*n* = 45)	25 (55.6%)	20 (44.4%)	**0.02**
Yes (*n* = 30)	8 (26.7%)	22 (73.3%)	

## Data Availability

The datasets generated during and/or analyzed during the current study are available from the corresponding author on reasonable request.

## References

[1] Stewart ST, Cutler DM, Rosen AB. Forecasting the effects of obesity and smoking on U.S. life expectancy. N Engl J Med 2009;361:2252–60. 19955525 10.1056/NEJMsa0900459 PMC4394736

[2] Nelson KM. The burden of obesity among a national probability sample of veterans. J Gen Intern Med 2006;21:915–9. 16918734 10.1111/j.1525-1497.2006.00526.x PMC1831589

[3] Younossi ZM, Stepanova M, Afendy M, Changes in the prevalence of the most common causes of chronic liver diseases in the United States from 1988 to 2008. Clin Gastroenterol Hepatol 2011;9:524–530.e1; quiz e60. 21440669 10.1016/j.cgh.2011.03.020

[4] Goldberg D, Ditah IC, Saeian K, Changes in the prevalence of hepatitis C virus infection, nonalcoholic steatohepatitis, and alcoholic liver disease among patients with cirrhosis or liver failure on the waitlist for liver transplantation. Gastroenterology 2017;152:1090–9.e1. 28088461 10.1053/j.gastro.2017.01.003 PMC5367965

[5] Sjöström L, Peltonen M, Jacobson P, Bariatric surgery and long-term cardiovascular events. JAMA 2012;307:56–65. 22215166 10.1001/jama.2011.1914

[6] Shreiner AB, Kao JY, Young VB. The gut microbiome in health and in disease. Curr Opin Gastroenterol 2015;31:69–75. 25394236 10.1097/MOG.0000000000000139 PMC4290017

[7] Zhu L, Baker SS, Gill C, Characterization of gut microbiomes in nonalcoholic steatohepatitis (NASH) patients: a connection between endogenous alcohol and NASH. Hepatology 2013;57:601–9. 23055155 10.1002/hep.26093

[8] Louis S, Tappu RM, Damms-Machado A, Huson DH, Bischoff SC. Characterization of the gut microbial community of obese patients following a weight-loss intervention using whole metagenome shotgun sequencing. PLoS One 2016;11:e0149564. 26919743 10.1371/journal.pone.0149564 PMC4769288

[9] Bajaj JS, Hylemon PB, Ridlon JM, Colonic mucosal microbiome differs from stool microbiome in cirrhosis and hepatic encephalopathy and is linked to cognition and inflammation. Am J Physiol Gastrointest Liver Physiol 2012;303:G675–85. 22821944 10.1152/ajpgi.00152.2012 PMC3468538

[10] Turnbaugh PJ, Hamady M, Yatsunenko T, A core gut microbiome in obese and lean twins. Nature 2009;457:480–4. 19043404 10.1038/nature07540 PMC2677729

[11] Dong TS, Katzka W, Lagishetty V, A microbial signature identifies advanced fibrosis in patients with chronic liver disease mainly due to NAFLD. Sci Rep 2020;10:2771. 32066758 10.1038/s41598-020-59535-w PMC7026172

[12] Million M, Lagier JC, Yahav D, Paul M. Gut bacterial microbiota and obesity. Clin Microbiol Infect 2013;19:305–13. 23452229 10.1111/1469-0691.12172

[13] Turnbaugh PJ, Ridaura VK, Faith JJ, Rey FE, Knight R, Gordon JI. The effect of diet on the human gut microbiome: a metagenomic analysis in humanized gnotobiotic mice. Sci Transl Med 2009;1:6ra14. 10.1126/scitranslmed.3000322 PMC2894525 20368178

[14] Dong TS, Jacobs JP. Nonalcoholic fatty liver disease and the gut microbiome: are bacteria responsible for fatty liver? Exp Biol Med (Maywood) 2019;244:408–18. 30871368 10.1177/1535370219836739 PMC6547005

[15] Jun BG, Park WY, Park EJ, A prospective comparative assessment of the accuracy of the FibroScan in evaluating liver steatosis. PLoS One 2017;12:e0182784. 28813448 10.1371/journal.pone.0182784 PMC5557594

[16] Grundy SM, Cleeman JI, Daniels SR, ; American Heart Association; National Heart; Lung; and Blood Institute. Diagnosis and management of the metabolic syndrome: an American Heart Association/National Heart, Lung, and Blood Institute Scientific Statement. Circulation 2005;112:2735–52. 16157765 10.1161/CIRCULATIONAHA.105.169404

[17] Dong TS, Luu K, Lagishetty V, A high protein calorie restriction diet alters the gut microbiome in obesity. Nutrients 2020;12:3221. 33096810 10.3390/nu12103221 PMC7590138

[18] Martino C, Morton JT, Marotz CA, A novel sparse compositional technique reveals microbial perturbations. mSystems 2019;4:e00016–19. 30801021 10.1128/mSystems.00016-19 PMC6372836

[19] Love MI, Huber W, Anders S. Moderated estimation of fold change and dispersion for RNA-seq data with DESeq2. Genome Biol 2014;15:550. 25516281 10.1186/s13059-014-0550-8 PMC4302049

[20] Adams LA, Waters OR, Knuiman MW, Elliott RR, Olynyk JK. NAFLD as a risk factor for the development of diabetes and the metabolic syndrome: an eleven-year follow-up study. Am J Gastroenterol 2009;104:861–7. 19293782 10.1038/ajg.2009.67

[21] Rosselli M, Lotersztajn S, Vizzutti F, Arena U, Pinzani M, Marra F. The metabolic syndrome and chronic liver disease. Curr Pharm Des 2014;20:5010–24. 24320032 10.2174/1381612819666131206111352

[22] Rich NE, Oji S, Mufti AR, Racial and ethnic disparities in nonalcoholic fatty liver disease prevalence, severity, and outcomes in the United States: a systematic review and meta-analysis. Clin Gastroenterol Hepatol 2018;16:198–210.e2. 28970148 10.1016/j.cgh.2017.09.041 PMC5794571

[23] Loomba R, Seguritan V, Li W, Gut microbiome-based metagenomic signature for non-invasive detection of advanced fibrosis in human nonalcoholic fatty liver disease. Cell Metab 2017;25:1054–62.e5. 28467925 10.1016/j.cmet.2017.04.001 PMC5502730

[24] Qiao S, Bao L, Wang K, Activation of a specific gut bacteroides-folate-liver axis benefits for the alleviation of nonalcoholic hepatic steatosis. Cell Rep 2020;32:108005. 32783933 10.1016/j.celrep.2020.108005

[25] Caussy C, Tripathi A, Humphrey G, A gut microbiome signature for cirrhosis due to nonalcoholic fatty liver disease. Nat Commun 2019;10:1406. 30926798 10.1038/s41467-019-09455-9 PMC6440960

[26] Qingxi-Zhao Hongyun-Wei. Characteristics of intestinal bacteria with fatty liver diseases and cirrhosis. Ann Hepatol 2019;18:796–803. 31558417 10.1016/j.aohep.2019.06.020

[27] Bashiardes S, Shapiro H, Rozin S, Shibolet O, Elinav E. Non-alcoholic fatty liver and the gut microbiota. Mol Metab 2016;5:782–94. 27617201 10.1016/j.molmet.2016.06.003 PMC5004228

[28] Buzzetti E, Pinzani M, Tsochatzis EA. The multiple-hit pathogenesis of non-alcoholic fatty liver disease (NAFLD). Metabolism 2016;65:1038–48. 26823198 10.1016/j.metabol.2015.12.012

[29] Caussy C, Hsu C, Lo MT, ; Genetics of NAFLD in Twins Consortium. Link between gut-microbiome derived metabolite and shared gene-effects with hepatic steatosis and fibrosis in NAFLD. Hepatology 2018;68:918–32. 29572891 10.1002/hep.29892 PMC6151296

[30] Da Silva HE, Teterina A, Comelli EM, Nonalcoholic fatty liver disease is associated with dysbiosis independent of body mass index and insulin resistance. Sci Rep 2018;8:1466. 29362454 10.1038/s41598-018-19753-9 PMC5780381

[31] Dao MC, Everard A, Aron-Wisnewsky J, ; MICRO-Obes Consortium. Akkermansia muciniphila and improved metabolic health during a dietary intervention in obesity: relationship with gut microbiome richness and ecology. Gut 2016;65:426–36. 26100928 10.1136/gutjnl-2014-308778

[32] Depommier C, Everard A, Druart C, Supplementation with Akkermansia muciniphila in overweight and obese human volunteers: a proof-of-concept exploratory study. Nat Med 2019;25:1096–103. 31263284 10.1038/s41591-019-0495-2 PMC6699990

[33] Everard A, Belzer C, Geurts L, Cross-talk between Akkermansia muciniphila and intestinal epithelium controls diet-induced obesity. Proc Natl Acad Sci U S A 2013;110:9066–71. 23671105 10.1073/pnas.1219451110 PMC3670398

[34] Plovier H, Everard A, Druart C, A purified membrane protein from Akkermansia muciniphila or the pasteurized bacterium improves metabolism in obese and diabetic mice. Nat Med 2017;23:107–13. 27892954 10.1038/nm.4236

[35] Dedrick S, Sundaresh B, Huang Q, The role of gut microbiota and environmental factors in type 1 diabetes pathogenesis. Front Endocrinol (Lausanne) 2020;11:78. 32174888 10.3389/fendo.2020.00078 PMC7057241

[36] Hasain Z, Mokhtar NM, Kamaruddin NA, Gut microbiota and gestational diabetes mellitus: a review of host-gut microbiota interactions and their therapeutic potential. Front Cell Infect Microbiol 2020;10:188. 32500037 10.3389/fcimb.2020.00188 PMC7243459

[37] Das SR, Kinsinger LS, Yancy WS Jr, Obesity prevalence among veterans at Veterans Affairs medical facilities. Am J Prev Med 2005;28:291–4. 15766618 10.1016/j.amepre.2004.12.007

[38] Bauer PV, Duca FA, Waise TMZ, Metformin alters upper small intestinal microbiota that impact a Glucose-SGLT1-Sensing glucoregulatory pathway. Cell Metab 2018;27:101–117.e5. 29056513 10.1016/j.cmet.2017.09.019

[39] Dong TS, Chang HH, Hauer M, Metformin alters the duodenal microbiome and decreases the incidence of pancreatic ductal adenocarcinoma promoted by diet-induced obesity. Am J Physiol Gastrointest Liver Physiol 2019;317:G763–72. 31545922 10.1152/ajpgi.00170.2019 PMC6962494

