# Supplementary figures and images for: Gut microbiome profiles associated with steatosis severity in metabolic associated fatty liver disease
Source: Hepatoma Res. Author manuscript; Available in PMC 2023 Jan 27. (PMC9881202; doi:10.20517/2394-5079.2021.55)

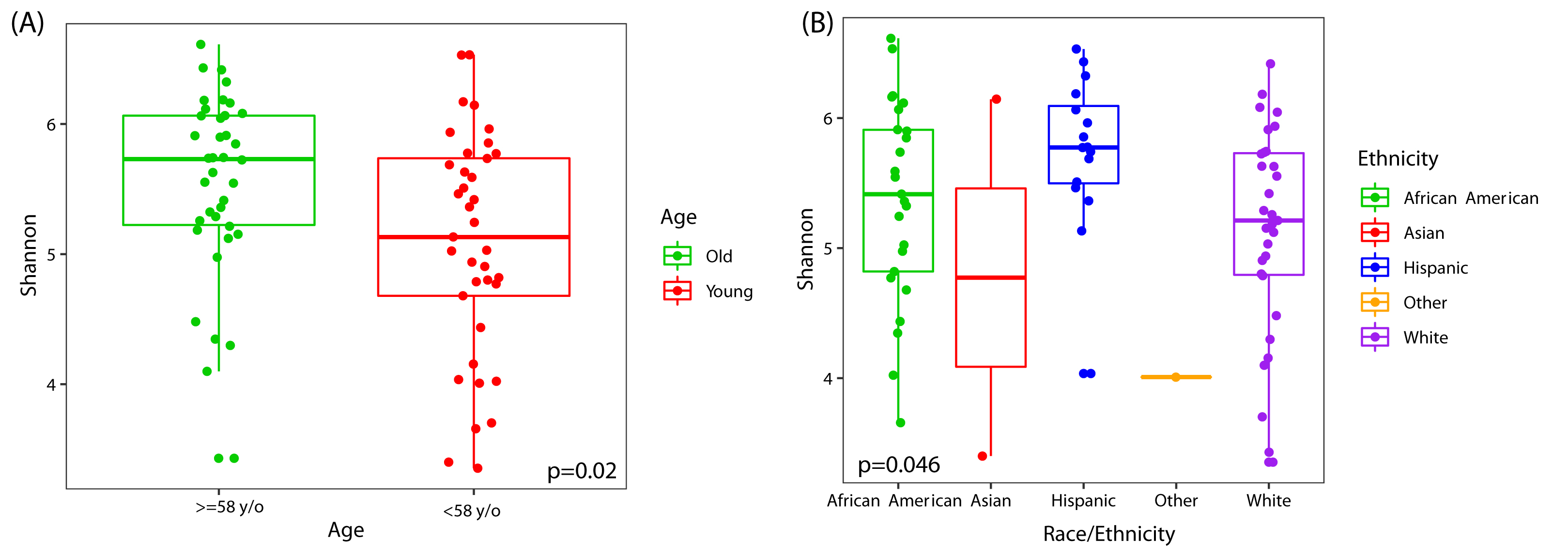

Supplement: Supplementary Figure 1 [file NIHMS1854425-supplement-Supplementary_Figure_1.jpg]
